# Molecular dissection and testing of PRSS37 function through LC–MS/MS and the generation of a PRSS37 humanized mouse model

**DOI:** 10.1038/s41598-023-37700-1

**Published:** 2023-07-14

**Authors:** Courtney Sutton, Kaori Nozawa, Katarzyna Kent, Alexander Saltzman, Mei Leng, Sureshbabu Nagarajan, Anna Malovannaya, Masahito Ikawa, Thomas X. Garcia, Martin M. Matzuk

**Affiliations:** 1 Center for Drug Discovery, Baylor College of Medicine, Houston, TX USA; 2 Department of Pathology & Immunology, Baylor College of Medicine, Houston, TX USA; 3 Mass Spectrometry Proteomics Core, Baylor College of Medicine, Houston, TX USA; 4 Verna and Marrs McLean Department of Biochemistry and Molecular Biology, Baylor College of Medicine, Houston, TX USA; 5 Department of Experimental Genome Research, Research Institute for Microbial Diseases, Osaka University, Suita, Osaka Japan; 6 The Institute of Medical Science, The University of Tokyo, Minato-Ku, Tokyo, Japan; 7 Scott Department of Urology, Baylor College of Medicine, Houston, TX USA

**Keywords:** Urogenital models, Experimental organisms, Genetic engineering, Mass spectrometry, Proteomic analysis

## Abstract

The quest for a non-hormonal male contraceptive pill for men still exists. Serine protease 37 (PRSS37) is a sperm-specific protein that when ablated in mice renders them sterile. In this study we sought to examine the molecular sequelae of PRSS37 loss to better understand its molecular function, and to determine whether human PRSS37 could rescue the sterility phenotype of knockout (KO) mice, allowing for a more appropriate model for drug molecule testing. To this end, we used CRISPR-EZ to create mice lacking the entire coding region of *Prss37*, used pronuclear injection to create transgenic mice expressing human PRSS37, intercrossed these lines to generate humanized mice, and performed LC–MS/MS of KO and control tissues to identify proteomic perturbances that could attribute a molecular function to PRSS37. We found that our newly generated *Prss37* KO mouse line is sterile, our human transgene rescues the sterility phenotype of KO mice, and our proteomics data not only yields novel insight into the proteome as it evolves along the male reproductive tract, but also demonstrates the proteins significantly influenced by PRSS37 loss. In summary, we report vast biological insight including insight into PRSS37 function and the generation of a novel tool for contraceptive evaluation.

## Introduction

Growing world populations are quickly displacing projected resource capacity and contraception is one clear way to help prevent unplanned births^1–3^. While multiple options exist for long-term reversible female contraception, a reversible non-hormonal male contraception is needed. Historically, contraception options, particularly for females, have consisted of hormone manipulation or a barrier method; however, a hormone-based contraception for males may not be a feasible option. While suppressing androgens can decrease sperm production, with variable results, it can also produce a myriad of other physiological changes that are not desirable, such as increases in aggressive behavior, libido, and weight, and decreases in cardiovascular health, lifespan, and testes size^4^. Therefore, a non-hormonal target for male contraception that does not induce these undesired physiologic consequences is needed. To this end, our logic is to target reproductive tract-specific proteins known to be required for male fertility since inhibiting these targets should lead to a reversible contraceptive effect with minimal side effects.

One such potentially druggable protein target is serine protease 37 (PRSS37), an inactive protease which is exclusively expressed in male germ cells during the late stages of spermatogenesis^5^, is required for sperm transit through the female reproductive tract and male fertility in mice^6,7^, and remains present on ejaculated human sperm^8^. PRSS37 interacts with and is required for proper processing of a disintegrin and metalloprotease 3 (ADAM3)^7,9,10^, which when deleted in male mice phenocopies PRSS37 KO males^6–9,11–14^. To identify the PRSS37 interactome, recent work by Xiong et al. utilizing a PRSS37-EGFP knock-in mouse and mass spectrometry has shown that PRSS37 can interact with calmegin (CLGN), calreticulin 3 (CALR3), protein disulfide-isomerase-like protein of the testis (PDILT), a disintegrin and metalloprotease 2 (ADAM2), a disintegrin and metalloprotease 4 (ADAM4), and a disintegrin and metalloprotease 6B (ADAM6B). From this work, it is theorized that PRSS37-PDILT-CALR3 function as a complex that interacts with, and is required for, ADAM3 processing^7^. Additionally, research conducted by Liu et al.^15^ compared men with unexplained male infertility (UMI) and men with proven fertility and found that men with UMI had lower PRSS37; and sperm with low PRSS37 had abnormal acrosin activation and premature proteolysis of ADAM2.

## Materials and methods

### Ethics statement

Mice were maintained in accordance with NIH guidelines, and all animal procedures were approved by the Institutional Animal Care and Use Committee (IACUC) at Baylor College of Medicine. This study was conducted and reported in accordance with ARRIVE guidelines^16^.

### Animals

Male and female B6D2F1 (C57BL/6 × DBA2) mice were purchased from Charles River (MA, USA). Timed-pregnant and pseudopregnant CD1 females were purchased from the Center for Comparative Medicine at Baylor College of Medicine (BCM). B6D2 mice from an in-house colony of intercrossed B6D2F1 mice were mated with PRSS37 F0 founder mice to expand the mutant line. For phenotypic analysis, sexually mature male mice were used. All mice were housed with a 12 h light cycle. All mouse experiments were performed according to the guidelines from the Institutional Animal Care and Use Committee at BCM (protocol AN-716).

### Generation of *Prss37* knockout mice

**Figure 1:**
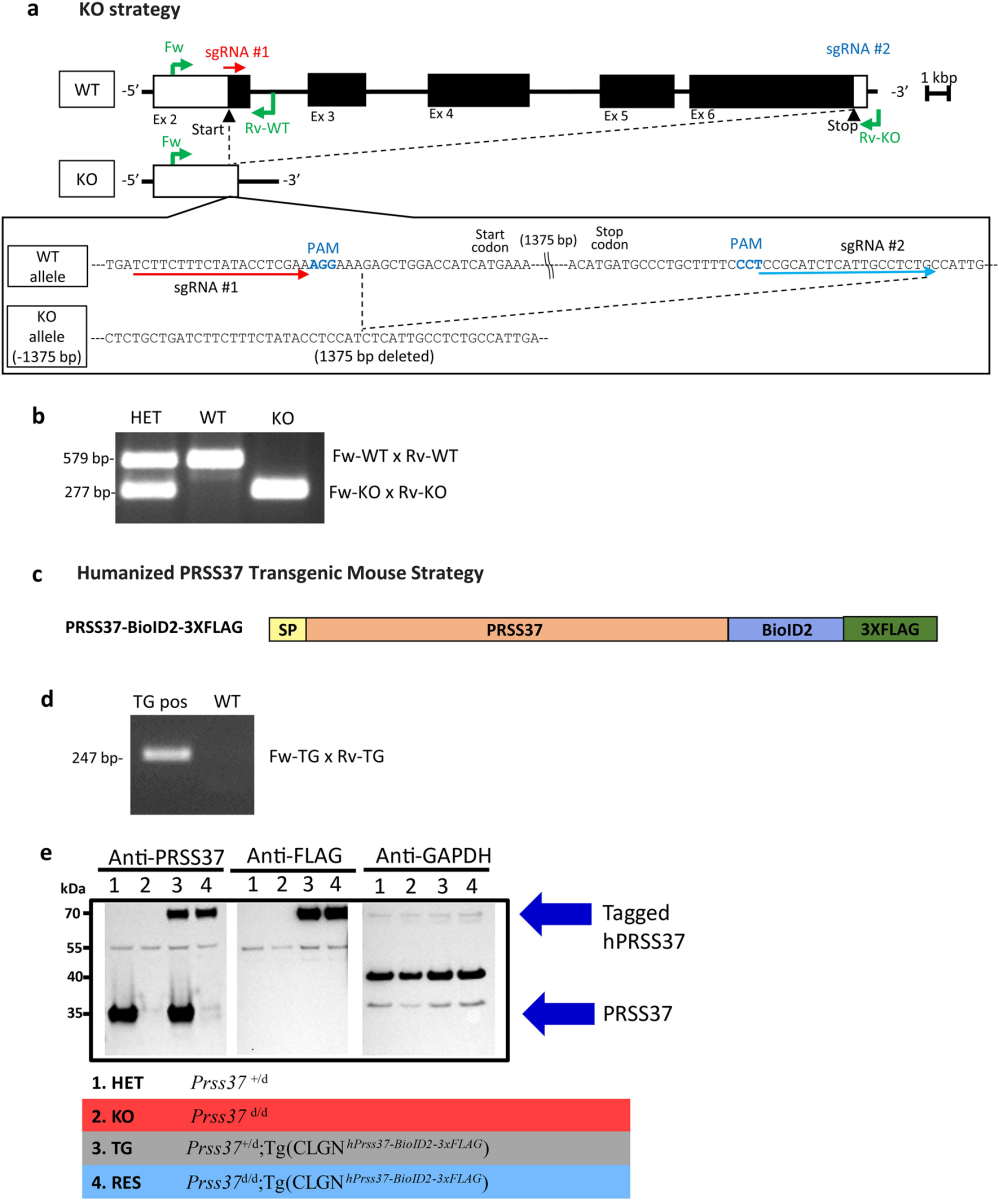
Developing *Prss37* mouse lines. (**a**) Genomic structure and strategy of generating KO mice of PRSS37, and the genetic sequences of mouse PRSS37 deleted by the CRISPR/Cas9 system. (**b**) Genotyping of PRSS37 alleles. Primers shown in (**b**) amplify specific amplicons for the WT (Fw-WT × Rv-WT), KO (Fw-KO × Rv-KO) alleles. This gel image was cropped so only the last 3 wells of the gel are present (lanes 14–16), the full gel can be found in the Supplementary Fig. S4. (**c**) Graphic of the humanized PRSS37 with the two identifying tags added at the C-terminus of the protein to generate the PRSS37 transgenic mouse line. (**d**) Genotyping of transgenic tag alleles. Primers shown in (**d**) amplify specific amplicons for the TG (Fw-TG × Rv-TG), alleles. This gel image was cropped so only lanes 3 and 4 of the gel are present, the full gel can be found in the Supplementary Fig. S4. (**e**) Immunoblotting to confirm expression of PRSS37 in HET, KO, TG, and RES mice in the first blot. With confirmatory blotting of Anti-FLAG in the transgenic mice in the second blot and Anti-GAPDH in all mice in the third blot. These blots were cropped so only bands between the molecular weight of 70 kDa and 35 kDa are shown, the full blots can be found in the Supplementary Fig. S4.

Single guide RNA (sgRNA) target sequences for mouse *Prss37* were designed (TCTTCTTTCTATACCTCGAAAGG and CCTCCGCATCTCATTGCCTCTG) using the CRISPRdirect suite (https://crispr.dbcls.jp/) (Fig. 1a). The custom gRNAs were ordered (Sigma) and assembled into a ribonucleoprotein (RNP) complex with Cas9 protein (Thermo Fisher Scientific) at 37 °C for 10 min. Oocytes were harvested from superovulated B6D2F1 females, in vitro fertilized with cauda epididymal sperm harvested from B6D2F1 males, incubated for 5–6 h, then electroporated with RNPs using an ECM 830 electroporation system (BTX, Holliston, MA). Embryos were cultured overnight to the 2-cell stage before being transferred into the oviducts of pseudopregnant CD1 mice. Founder mutations in pups born were identified by PCR and Sanger sequencing. Three founder females with the same − 1375 bp deletion were used to expand the colony and generate *Prss37*^*d/d*^ (KO) male and female mice. Mice were genotyped by PCR with specific primers for the wild-type (WT) alleles (Fw-WT: 5′-GGAGCTCCTCTTCCCAAACA-3′ and Rv-WT: 5′-AGGGTTCTACAGGGCAGGTA-3′) or KO alleles (Fw-KO: 5′-CTCCACCTGCTGATTTTGCC-3′ and Rv-KO: 5′- GCAGCCCATTTCTTCCCATC-3′) (Fig. 1b, Supplementary Fig. S4).

### Generation of humanized *Prss37* transgenic mice

The endogenous coding sequence of human *PRSS37* (ENST00000350549.8) was PCR amplified to remove the stop codon, and to introduce 5′ and 3′ XbaI and HindIII sites, respectively, for ligation into pCAG-MCS-BioID2-3xFLAG (Addgene plasmid #186812)^17^.The resulting protein expressed from this vector is a fusion of human PRSS37 at the N-terminus and BioID2-3xFLAG tag at the C-terminus (Fig. 1c). After confirmation of protein expression at the appropriate molecular weight in HEK293 cells, the CAG promoter sequence was replaced with the mouse *Clgn* promoter to drive spermatid-specific, and not ubiquitous, expression of the transgene, as previously described^13,17^. After large-scale purification of our custom pCLGN-hPRSS37-BioID2-3xFLAG plasmid, the linearized product was delivered to the Genetically Engineered Rodent Models (GERM) Core at BCM^18^ where the transgene was injected into the pronucleus of fertilized eggs (n = 40), and then transferred into the oviduct ampulla of pseudopregnant CD1 females (n = 8). The resulting founder pups were genotyped for the presence of the complete transgene through PCR and Sanger sequencing (Fig. 1d, Supplementary Figs. S1, S4). For subsequent genotyping, primers within the BioID2 tag were used to identify the presence of the transgene, as follows: Fw-TG: 5′-TGAAGGAGTGGAACGTGAGC-3′ and Rv-TG: 5′-TGGAAGTACACGTCGTTGGG-3′. *Prss37* KO females were intercrossed with male mice harboring the hPRSS37-BioID2-3xFLAG transgene without or with a single *Prss37* null allele to produce transgenic (TG) control [*Prss37*^+/d^;Tg(CLGN^*hPrss37-BioID2-3xFLAG*^)] and rescue (RES) [*Prss37*^d/d^;Tg(CLGN^*hPrss37*-*BioID2-3xFLAG*^)] male mice, respectively, as shown in Supplementary Fig. S1. Genotyping of all null and transgenic alleles were confirmed before any mating cages were set up for fertility testing.

### Male fertility assessment

Sexually mature KO, TG, and RES male mice were continuously housed with two C57BL6J/129SvEv female mice per male for 4.5 months. To perform fertility testing and obtain additional KO mice, HET male mice were housed with HET female mice during the same period. During the mating period, the number of pups born per litter per male was counted. The total number of litters and pups per male over the mating trial was calculated and divided by the number of months to generate averages and statistics per genotype. Unless an entire group of males displayed sterility (for all males tested of a given genotype, no pups were sired during the entire mating period), the average number of pups per litter is based on the average litter size per male where a litter is defined as one or more pups.

### Protein extraction and Immunoblotting

Testicular germ cells (TGCs) from adult male mice were homogenized using VWR Bead Mill Homogenizer in Pierce IP buffer containing protease inhibitor followed by a 30-min rest on ice. Lysates were cleared by centrifugation at 15,000× *g* for 15 min at 4 °C. Supernatants were collected, mixed with NuPAGE LDS Sample Buffer (4×) (ThermoFisher Scientific, Waltham, MA, USA) to 1× final concentration and 2-Mercaptoethanol to 2.5% final concentration, denatured at 95 °C for 5 min, subjected to SDS-PAGE, transfer to nitrocellulose membrane, then blocking of the membrane with Bullet Blocking One (Nacalai USA). The samples were then evaluated by immunoblotting with antibodies against PRSS37 (1:500 dilution; Sigma, St. Louis, MO, USA), FLAG (HRP, 1:1000, Sigma, St. Louis, MO, USA), and GAPDH (HRP, 1:5000, Proteintech, Rosemont, IL, USA) overnight at 4 °C (Fig. 1e, Supplementary Fig. S4). The chemiluminescent signal was developed using Pierce™ ECL Western Blotting Substrate and iBright Imaging System (ThermoFisher Scientific, Waltham, MA, USA) at an exposure time of 5 s.

### Computer-assisted sperm analysis (CASA)

Sperm were extracted by mincing the cauda of the epididymis 30 times with dissection scissors in 1 mL of Enhance Sperm Wash w/Gentamicin (Vitrolife, Sweden) medium. All media, pre- and post-addition of sperm, was preincubated and maintained at 37 °C throughout the procedure. After incubation for 15 min—with a single end-over-end inversion of the tube midway during the incubation, to facilitate release of the sperm from the tissue fragments—50 µL of the supernatant was added to a fresh aliquot of 950 µL of medium, mixed gently, then approximately 6 µL of diluted sample was applied via capillary action into a single chamber of a dual chambered 20 µm-depth Leja semen analysis slide (Spectrum Technologies, Healdsburg, CA). Sperm parameters were measured using the Hamilton Thorne CEROS II system. Per sample, a minimum total of 200 sperm were measured, which typically consisted of recording a minimum of five non-overlapping fields. The remaining sample was maintained for an additional 90 min to allow for capacitation, changes in hyperactivation, and remeasurement. The CASA instrument provided measurements of the following parameters: sperm count (M/mL), sperm motility (%), progressive cells (%), static cells (%), hyperactivation (%), average path velocity (VAP; µm/sec), curvilinear velocity (VCL; µm/sec), progressive velocity (VSL; µm/sec), mean amplitude of lateral head displacement (ALH; µm), beat cross frequency (BCF; Hz), linear coefficient (LIN = VSL/VCL; %), straightness (STR = VSL/VAP; %), and wobble (WOB = VAP/VCL; %).

### Tissue processing for proteomics profiling

To investigate PRSS37 protein in vivo, we conducted proteomic profiling using testis germ cells, and epididymis sections (caput, corpus, and cauda) from two mice each of *Prss37* HET and KO lines. Each animal’s left and right sections of the reproductive tract were used as its own replicate and in total 32 samples were sent to the Mass Spectrometry Proteomics Core at BCM for profiling. Briefly, the male reproductive tract was collected from the mice and fat was trimmed away from the epididymis and then the epididymis was cut into 3 different sections: caput, corpus, and cauda. Each of the testes were collected, washed, and detunicated, then only the seminiferous tubules cut and carefully placed in 40 mL of 1× PBS with 1 mg/mL BSA (Millipore Sigma, Burlington, MA). The tube was kept on a rocker at room temperature for 7 min to facilitate unraveling of the seminiferous tubules and release of interstitial cells. Next, to facilitate further dispersion of the seminiferous tubules, the sample was passed back-and-forth through a 10 mL serological pipette for approximately 3 min. Seminiferous tubule fragments were allowed to sediment at 1× *g* for 3 min, then washed 3 times with 40 mL 1× PBS. On a fourth wash in 1 mL volume, the seminiferous tubules were transferred to a 1.5 mL tube, sedimented completely, PBS removed, stored at − 80 until they were delivered to the Mass Spectrometry Proteomics Core at BCM for all subsequent processing. The sample preparation for proteome profiling including protein lysis and digestion, peptide desalting, and offline fractionation was similar as described before^19^. The peptides were labeled using TMTpro16plex Label Reagent Set (Thermo Scientific A44522) according to the manufacturer’s protocol. The TMTpro 16plex channel assignment was as follows: the 16 right sections of the reproductive tract were assigned to one TMTpro16plex: Prss37 KO right section tissues-126, 127N, 127C, 128N, 128C, 129N, 129C, 130N, Prss37 Het right section tissues-130C, 131N, 131C, 132N, 132C, 133N, 133C, 134; the 16 left sections of the reproductive tract were assigned to another TMTpro16plex: Prss37 KO left section tissues-126, 127N, 127C, 128N, 128C, 129N, 129C, 130N, Prss37 Het left section tissues-130C, 131N, 131C, 132N, 132C, 133N, 133C, 134.

The peptides were analyzed using Vanquish Neo UHPLC system (Thermo Fisher Scientific, San Jose, CA) coupled to Orbitrap Eclipse mass spectrometer (Thermo Fisher Scientific, San Jose, CA). 250 ng of each fraction for proteome was loaded on Pepmap Neo trap (5 μm × 300 μm × 5 mm, C18) switched in-line with an in-housed 20 cm × 75µmI.D. column (Reprosil-Pur Basic C18, 1.9 µm, Dr.Maisch GmbH, Germany). Peptide separation was done at a flow rate of 250 nl/min over 110-min gradient time with different concentrations of solvent B (2–30% 88 min, 30–60% 6 min, 60–90% 7 min, and finally hold at 50% 7 min). The peptides were ionized at positive spray voltage (2.4 kV) and the ion transfer tube temperature was 300 °C. The mass spectrometer was operated in a data dependent mode with 2 s cycle time. The MS1 was done in Orbitrap (120,000 resolution, scan range 400–1600 m/z, standard AGC, auto Injection time) followed by MS2 in Orbitrap (30,000 resolution, AGC 1e5, 54 ms injection time, HCD 38%) with TurboTMT algorithm. Dynamic exclusion was set to 20 s and the isolation width was set to 0.7 m/z. The data processing and analysis was carried out as described before^19^ with an exception that no phosphoproteomics analysis was conducted in the current study.

### Proteomics profiling

Proteomics raw data files were converted to mzML using MSConvert^20^. MASIC was used to extract the precursor ion intensities for each peptide via area under elution curve as well as the reporter ions intensities^21^. Butterworth smoothing method was used with sampling frequency of 0.25 and an SCI tolerance of 10 ppm. Reporter ion tolerance was set to 0.003 Da with reporter ion abundance correction enabled. The mouse RefSeq database was downloaded on 2020-03-09 (identifier CF_000001635.26_GRCm38.p6_prtoein) and reversed decoys and common contaminants were added using Philosopher^22^. Raw spectra were searched with MSFragger (v3.5), ran with mass calibration^23,24^. Settings included strict trypsin digestion for amnio acids from 7 to 50 amino acids with up to two missed cleavages, clip protein N-term methionine, precursors ions with charge 2–6, precursor mass mode set to CORRECTED, isotope error set to − 1/0/1/2. For fragment spectra, TopN was set at 150 and the mass range cleared between 125 and 135 to filter out reporter ions during spectral matching. A precursor mass error was set at ± 20 ppm and a fragment mass tolerance of ± 0.02 Da. Static modification of C + 57 carbamidomethylation and peptide N-terminus TMT16 label + 304.207. Dynamic modifications methionine oxidation (+ 15.9949), acetylation (+ 42.0106), peptide N-terminal TMT16 label (+ 304.207), peptide N-term acetylation, and peptide N-term pyroGlu (− 17.02650 for Q,C) and (− 18.01060 for E). Multiple variable modifications were allowed. Peptide validation was performed using semi-supervised learning procedure in Percolator^25^ as implemented in MokaPot^26^. Peptides were grouped and quantified into gene product groups using gpGrouper^27^. Gene products identified in both multiplexes were retained for downstream analysis. Resulting protein values were median normalizes and log transformed, then batch corrected across multiplexes using ComBat^28^ as implemented in the Surrogate Variable Analysis package (SVA package R version 3.44.0)^29^. Heatmaps were generated with ComplexHeatmap^30^. Data wrangling performed with Python 3.8 (Python Software Foundation. Python Language Reference http://www.python.org), along with third-party libraries Numpy^31^ and Pandas (mckinney-proc-scipy-2010, reback2020pandas). All proteomic experiments are available in Massive/ProteomeXchange with the following accession number: MSV000091662.

### Statistical analysis

Statistical significance was evaluated using the two-tailed unpaired Student t-test assuming unequal variances except as otherwise noted. In analyses involving 2 or more groups, a One-Way ANOVA was used to determine differences between groups (Prism 9.5.1, Boston, MA, USA). For proteomics data, following batch corrections across multiplexes, group differences were assessed using the moderated t-test as implemented in the R package limma (R version 4.2)^32^, and false discovery rate controlled with the Benjamini–Hochberg procedure^33^. Data are represented as means ± SEM. *P < 0.05, ns, not significant.

## Results

### Generation of *Prss37* KO mice

PRSS37 was previously identified by Shen et al. in 2013 as a conserved testis-specific gene required for male mouse fertility^6^. To confirm their result, and to perform proteomics experiments needed to help further decipher the critical role of PRSS37 in spermatozoa function, we generated a new *Prss37* KO mouse model using the CRISPR/Cas9 system in which we deleted the entire coding sequence of *Prss37*. The simultaneous use of two single guide RNAs (sgRNAs), each flanking nearly the entire protein coding region of mouse *Prss37* (Fig. 1a), resulted in a desired − 1375 bp deletion, which was confirmed by PCR and Sanger sequencing (Fig. 1b, Supplementary Fig. S1). Representative genotyping results for WT, HET, and KO mice are presented in Fig. 1a. The absence of PRSS37 protein (and demonstration that the new allele is null) was confirmed by Western blot analysis using anti-PRSS37 antibody (Fig. 1e). The KO mice did not show any obvious developmental abnormalities or differences in sexual behavior.

### Prss37 KO males are sterile

**Figure 2:**
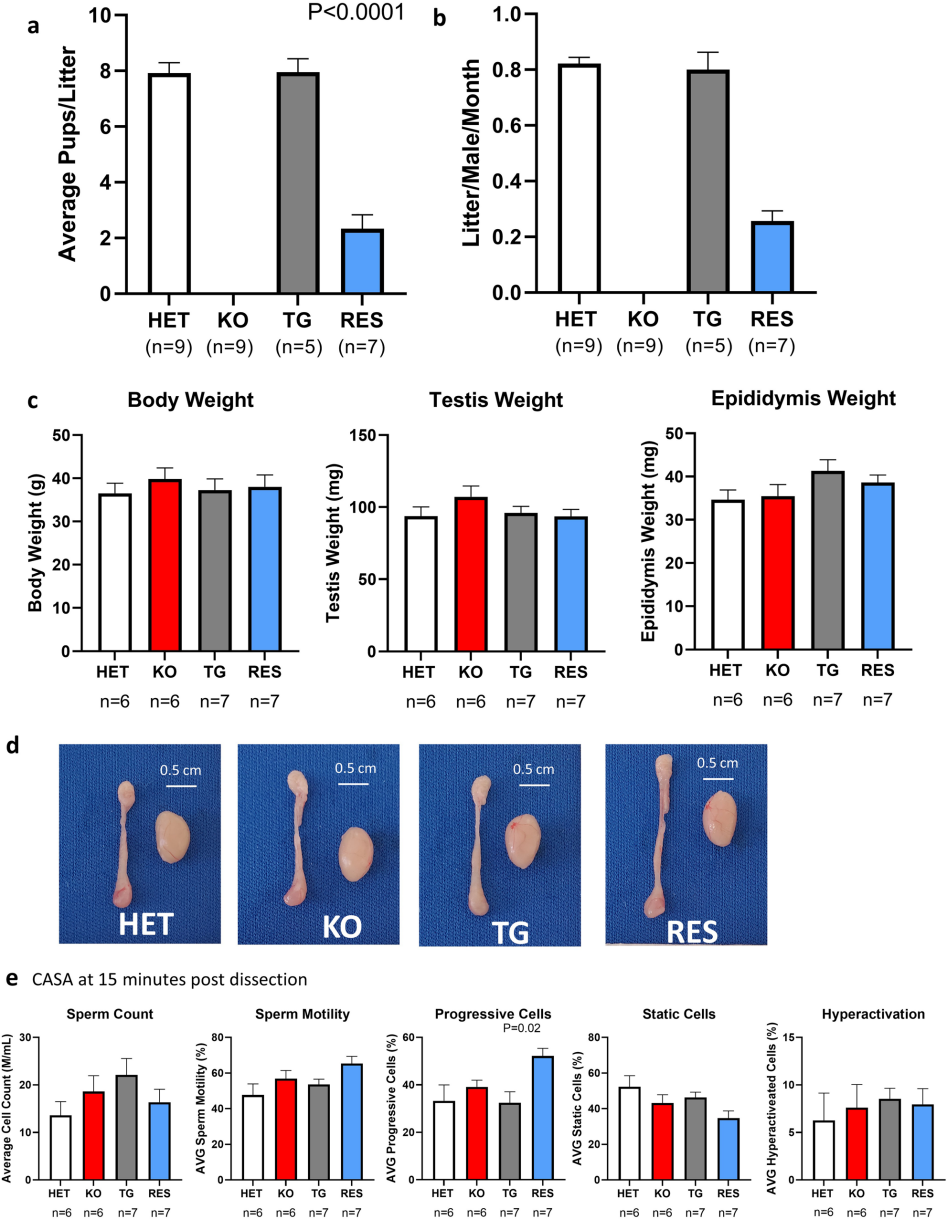
*Prss37* transgenic mice can rescue fertility in *Prss37* KO mice. (**a**) Average litter size from natural mating of *Prss37* HET, KO, TG, and RES mice. Litter size was measured by the number of pups born. *Prss37* KO males were infertile, *P* < 0.0001. (**b**) Average litter per male per month, from natural mating of *Prss37* HET, KO, TG, and RES mice. Litter size was measured by the number of pups born. *Prss37* RES males were able to rescue fertility, in endogenous *Prss37* KO males. (**c**) The average body weight, testis weight, and epididymis weight from *Prss37* HET, KO, TG and RES mice. (**d**) Images of *Prss37* HET, KO, TG and RES testis and epididymis. Scale bar, 5 cm. (**e**) CASA parameters between *Prss37* HET, KO, TG, and RES mice looking at Time point at 15 post dissection for sperm count, sperm motility, progressive cells, static cells, and hyperactivation.

Sexually mature male *Prss37* KO and HET mice were housed with females for 4.5 months with a detailed log of every litter produced. While HET males (*N* = 9) sired 0.82 ± 0.02 litters/month with 7.9 ± 0.77 pups/litter (Fig. 2a,b), KO males (*N* = 9) sired no litters or pups during the same time period (Fig. 2a,b), confirming that PRSS37 is required for male fertility. TG males (*N* = 5) sired 0.80 ± 0.06 litters/month and 7.95 ± 0.97 pups/litter (Fig. 2a,b), demonstrating that the introduction of the human PRSS37 transgene does not produce a deleterious gain-of-function effect on fertility, while RES males (*N* = 7) could sire 0.25 ± 0.03 litters/month and 2.3 ± 0.56 pups/litter (Fig. 2a,b), indicating that the human transgene, while not fully restoring fertility to baseline levels, can partially restore fertility. Based on these results, our humanized PRSS37 transgenic mouse may serve as model for small molecule testing.

We also examined phenotypic attributes to each of our mouse lines to determine if there was a difference in reproductive tissue and body weight. We found that there was no statistical difference in body weight (HET = 36.5 ± 2.4 g, KO = 39.8 ± 2.6 g, TG = 37.3 ± 2.6 g, RES = 38.0 ± 2.7 g) (Fig. 2c), testis weight (HET = 93.6 ± 6.5 mg, KO = 107.2 ± 7.5 mg, TG = 95.9 ± 4.7 mg, RES = 93.5 ± 4.9 mg) (Fig. 2c), or epididymis weight (HET = 34.7 ± 2.2 mg, KO = 35.5 ± 2.7 mg, TG = 41.3 ± 2.6 mg, RES = 38.6 ± 1.7 mg) between any of the mouse line(Fig. 2c). Additionally, we detected no differences in testis or epididymis morphology across the different lines (Fig. 2d).

### CASA

To investigate sperm parameters, before and after capacitation/hyperactivation, across all four (HET, KO, TG, and RES) mouse lines, we performed CASA at 15 and 90 min after dissection, respectively. At 15 min, sperm from RES males displayed a statistically significant (*P* = 0.02) increase in percent of progressive cells compared to other lines (Fig. 2e). However, at 90 min this increase disappeared (Supplementary Fig. S2). Of all the other parameters measured—count, % motility, % static cells, hyperactivation, VAP, VCL, VSL, ALH, BCF, LIN, STR, and WOB—no statistically significant differences could be found across any of the four lines and at either time point (Fig. 2e, Supplementary Fig. S2). Based on these findings, sperm concentration and activity appear normal in RES males, adding further support to the utility of this mouse model.

### Proteomics profiling

**Figure 3:**
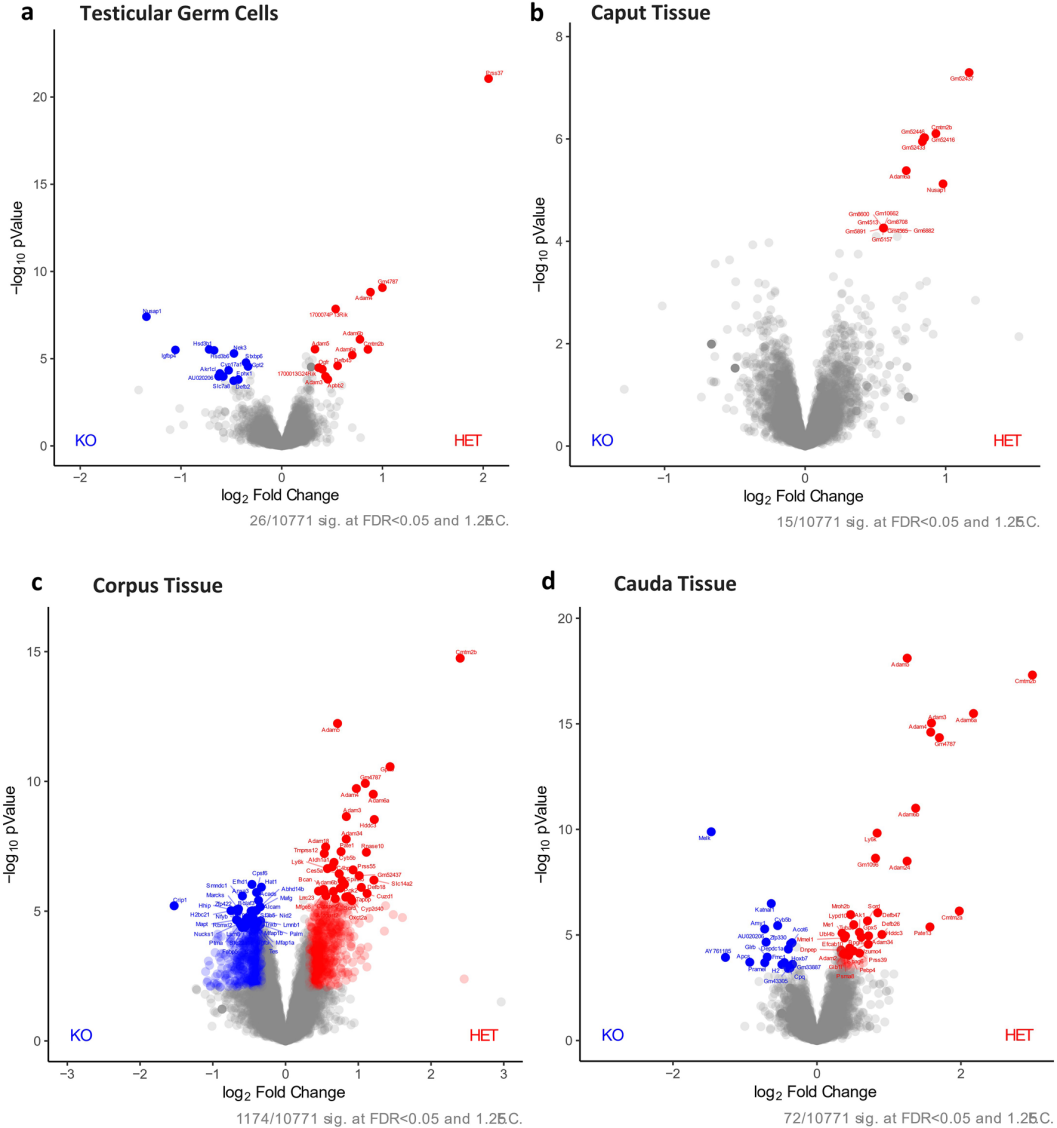
Protein expression in *Prss37* HET and *Prss37* KO Mice from proteomics profiling. (**a**) Volcano plot of quantitative analysis of proteins in testicular germ cells identified by MS profiling. Proteins expressed with fold-changes (HET: KO ratio) > 1.25. (*P* < 0.05) were selected. (**b**) Volcano plot of quantitative analysis of proteins in the caput epididymis identified by MS profiling. Proteins expressed with fold-changes (HET: KO ratio) > 1.25. (*P* < 0.05) were selected. (**c**) Volcano plot of quantitative analysis of proteins in the corpus epididymis identified by MS profiling. Proteins expressed with fold-changes (HET: KO ratio) > 1.25. (*P* < 0.05) were selected. (**d**) Volcano plot of quantitative analysis of proteins in the cauda epididymis identified by MS profiling. Proteins expressed with fold-changes (HET: KO ratio) > 1.25. (*P* < 0.05) were selected.

**Figure 4:**
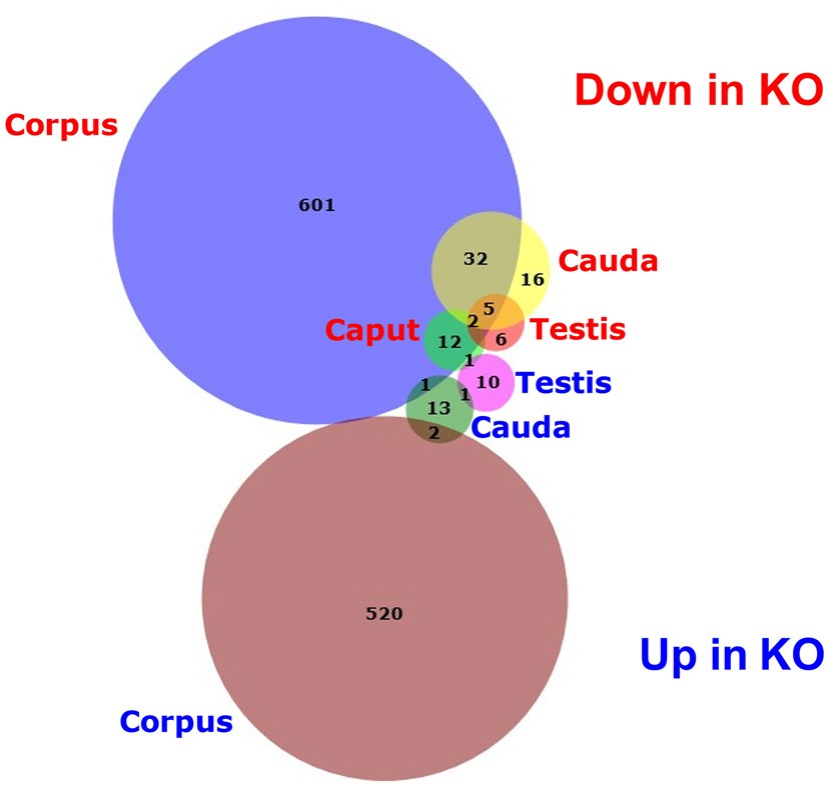
Venn diagram of protein expression. This Venn Diagram shows the how different proteins have increased expression or decreased expression in the *Prss37* KO mice. It also illustrates how an abundant change in protein expression is occurring in the corpus epididymis compared to any other tissue section regardless of whether it was increasing or decreasing in the KO mouse.

**Figure 5:**
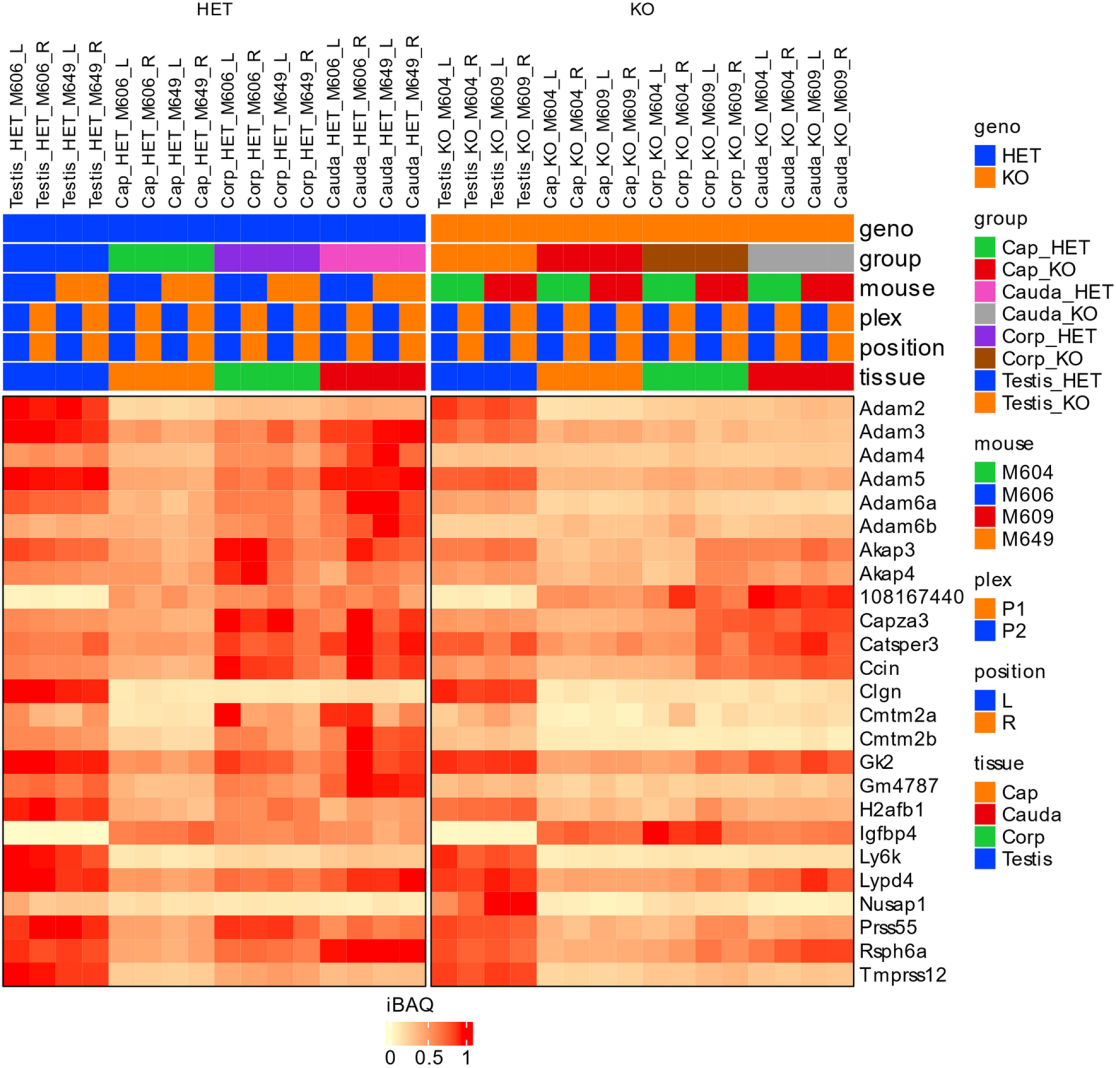
Heat map with characterizing protein abundance in proteins that are known to be important for reproductive success.

To detect potential sperm protein differences in the absence of PRSS37, we performed proteomic analysis of male reproductive tissues (testes and regions of the epididymides) from control (HET) and KO adult male mice. In quantitative profiling, 10,771 proteins were detected across our HET and KO tissues. With our filtering parameters set at fold change (FC) greater than 1.25 (or less than − 1.25) and false detection rate (FDR) less than 0.05, there were 1224 proteins differentially present in HET versus KO across all tissues analyzed (testis, caput, corpus, cauda). Consistent with our KO being a null allele lacking PRSS37, PRSS37 was found to be the most depleted protein in the KO testes (Fig. 3a). Twenty-six proteins were differentially present in testis (13 significantly increased in KO, 13 significantly decreased in KO) and 1208 were differentially present in one or more segments of the epididymides: 15 proteins in caput (15 significantly increased in KO, 0 significantly decreased in KO) (Fig. 3b), 1179 proteins in corpus (655 significantly increased in KO, 524 significantly decreased in KO) (Fig. 3c), and 73 proteins in cauda (55 significantly increased in KO, 18 significantly decreased in KO) (Fig. 3d). Fifty-eight proteins were found significantly altered in two, three, or all four tissues as indicated in the Venn diagram in Fig. 4, such as ADAM24 and ADAM34 that were found decreased in both KO corpus and KO cauda (Fig. 4, Supplementary Table S1) and APCS and AY761185 that were found increased in both KO corpus and KO cauda (Fig. 4, Supplementary Table S1). Of the 26 proteins differentially present in testis, seven proteins (ADAM3, ADAM4, ADAM5, ADAM6A, ADAM6B, CMTM2B, GM4787) were significantly decreased in KO testis while either significantly decreased in all three segments of KO epididymides (ADAM6A and CMTM2B) or significantly decreased in both corpus and cauda of the KO (ADAM3, ADAM4, ADAM5, ADAM6B, GM4787); two proteins (AU020206 and IGFBP4) were significantly increased in KO testis while significantly increased in KO cauda (AU020206) or KO corpus (IGFBP4); and 1 protein (NUSAP1) was significantly increased in KO testis while significantly decreased in KO caput (Fig. 5, Supplementary Table S1). Interestingly, ADAM3, ADAM5, ADAM6A, ADAM6B, CMTM2B, and GM4787 are either testis-specific or -enriched proteins^5^, and of these, ADAM3 and ADAM5, when ablated in KO animal models, display male infertility phenotype^34–37^. Of the remaining significantly increased or decreased proteins that are male reproductive tract-specific with infertile mouse models, 8 are testis-specific genes found significantly decreased in KO corpus (AKAP3, AKAP4, CAPZA3, CATSPER3, CCIN, GK2, H2AL2A, and RSPH6A) and 1 is a dual testis/epididymis-specific gene (OAZ3).

From previous research done by our lab and collaborators^5–14^, we know that certain testis-specific proteins are important for sperm migration through the uterotubal junction (UTJ), and therefore required for successful fertilization of the oocyte. We therefore determined the effect of PRSS37 absence on these proteins in our dataset (Fig. 4). As mentioned above, ADAM3, ADAM4, and CMTMB2 were significantly decreased in KO testis and either two or all three segments of KO epididymis (Fig. 5, Supplementary Fig. S3, Supplementary Table S1). In addition, ADAM2 was significantly decreased in KO corpus and cauda (Fig. 5, Supplementary Fig. S3), CMTM2A and LY6K were significantly decreased in KO corpus and KO cauda (Fig. 5, Supplementary Table S1, Supplementary Fig. S3), and LYPD4, PRSS55, and TMPRSS12 were significantly decreased in KO corpus (Fig. 5, Supplementary Fig. S3). Surprisingly, CLGN was significantly increased in KO corpus (Fig. 5, Supplementary Fig. S3). This indicates that PRSS37 is critically involved in the testicular and/or epididymal stabilization and/or processing of these proteins.

## Discussion

Hormonal options for male contraception offer a wide array of negative side effects that would likely dissuade users from using it, therefore making a non-hormonal option a more appealing contraceptive choice. A main objective of the research conducted in our laboratories is to identify viable targets for non-hormonal contraception that could go a long way to providing reproductive rights for men. Using our large deletion *Prss37* KO mouse line, we confirmed that PRSS37 is essential for male fertility when comparing results from fertility tests with HET and KO males (Fig. 2a). Previous research conducted by Shen et al.^6^ used manipulation of ES cells to generate a *Prss37* KO mouse. By replacing Exon 3 and Exon 4 with a PGK-Neo cassette, their deletion resulted in less coding region ablated than our model, which removed all genetic material between Exon 2 and Exon 6 (Fig. 1a). While Shen et al. also showed that *Prss37* KO mice were infertile, they conducted their experiment over only a brief 48 h time period where males were continuously mated, and, if the presence of a plug was found, females were removed and replaced with new females, and removed females with evidence of plug formation were kept until they produced a litter^6^. In contrast, our fertility study lasted for 4.5 months to include the average number of pups per litter and the average number of litters per male, adding stronger evidence of a sterility phenotype. Furthermore, our study is the first to demonstrate that mouse PRSS37 protein can be functionally replaced with the human protein sequence, allowing for a more appropriate model for human drug testing (Fig. 2a). Taken together, these results demonstrate that PRSS37 is a strong viable candidate for the development of non-hormonal male contraception. Several of our projects have looked at blocking PRSS37 through different means, as universally disrupting this protein can result in infertility with sperm being unable to bind to the zona pellucida (ZP)^6,7,15^. Trying to rescue fertility in the mouse model by generating the RES line provides another key component in determining contraceptive viability. Given that we were able to restore fertility, we were able to show that we could reverse the effects of removing endogenous PRSS37 from the mouse and using a humanized PRSS37 in its place. One of the major challenges of hormonal contraception in males is that blocking testosterone or DHT also comes with the complication of removing the major hormones responsible for muscle building and testicular size^38,39^. No differences in body or reproductive tract weight were noted, further solidifying PRSS37 as a strong candidate for non-hormonal contraception (Fig. 2c,d). The CASA data at 15 min post dissection RES mice displayed similar sperm characteristics to all other groups, apart from RES having an increased number of progressive cells (Fig. 2e). These results are expected since sperm lacking PRSS37 fail to traverse the female reproductive tract and successfully cross the UTJ without affecting sperm parameters. Shen et al.^6^ conducted their CASA analysis after a 5 min incubation, we tried to match more physiological conditions, using a 15 min incubation to allow sperm to swim up and out of the cut portion of the cauda epididymis and again at 90 min to simulation capacitation of the sperm cells.

The proteomics profiling conducted on the KO and HET mice provided several reproductive tract specific proteins that might prove to be additional valuable contraceptive targets (Fig. 5). Additional proteins of interest to our lab, PRSS55 and TMPRSS12, have a similar pattern of protein abundance between our KO and HET as PRSS37 does, this indicates PRSS37 may have been activating or working concurrently with additional proteases in some type of signal cascade (Fig. 5, Supplementary Fig. S3). This down regulation of other reproductive tract serine proteases could indicate some mechanism action that PRSS37 is acting upstream or downstream of these proteins to enable ample production. These proteins will require further examination since the disruption of PRSS37 clearly disrupted abundance of other proteins, these protein cascades will have to be examined thoroughly for overlap. Previous research has shown that ADAM3 is important for sperm binding to the ZP and downstream protein activation^6,7,9,10,12–14,40,41^. Based on our results, it is possible that there are more interactions between the PRSS37 than just ADAM3.

These results have shown similarities between the proteomics results generated by Xiong et al.^7^ and their GFP-IP/MS strategy using protein lysates from testis. Our TGCs results also showed that PDILT was important in the PRSS37 signal cascade, but our proteomics research indicated that ADAM3, and CMTM2B had a higher abundance, compared to Xiong et al.^7^ who observed ADAM2 and CALR3 proteins. Our proteomics research also indicates other downstream protein targets that could be affected by the absence of PRSS37, and some potential new targets to explore for male contraception.

**Figure 6:**
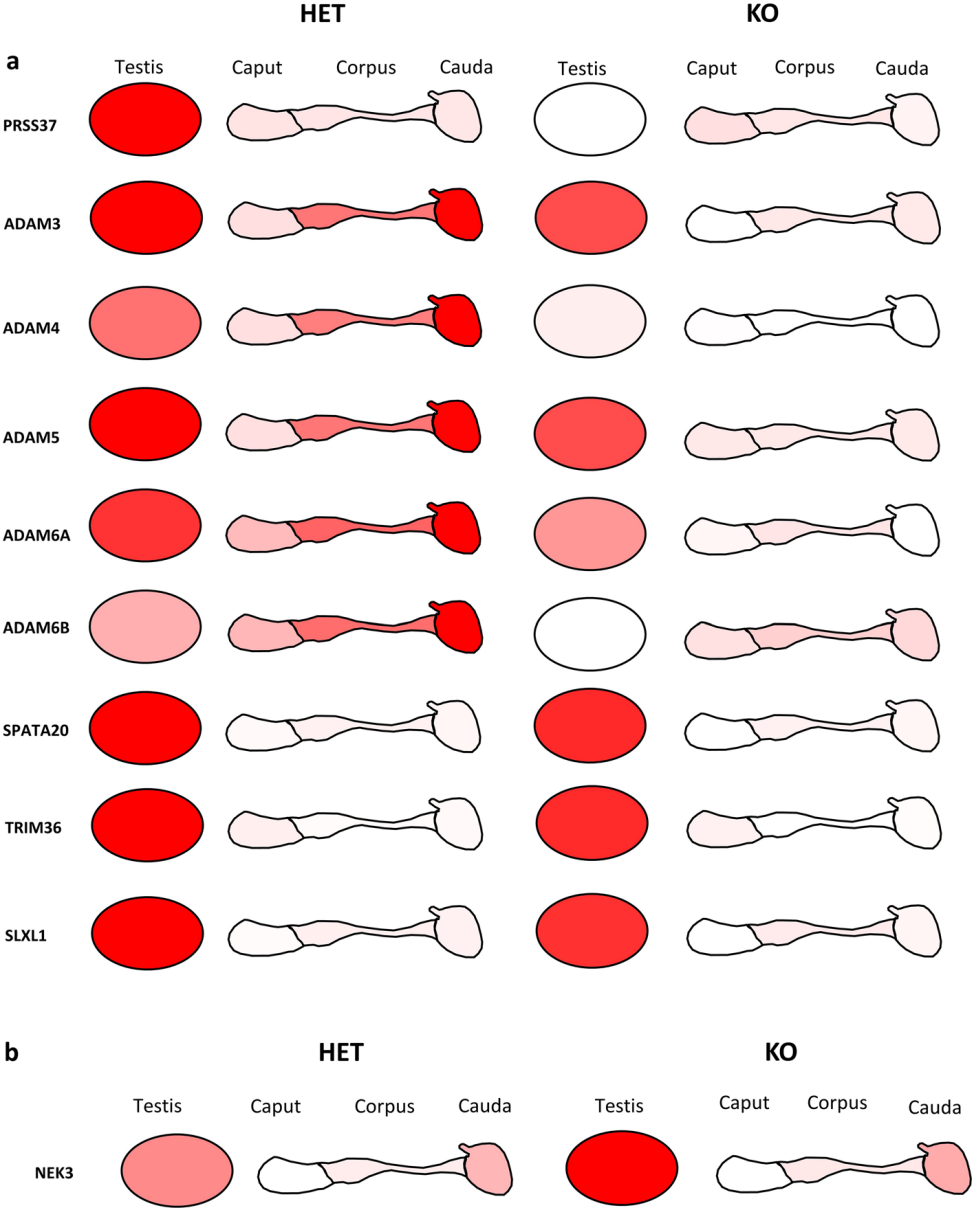
Graphical heat map of proteins that are differentially expressed between HET and KO mice. (**a**) Nine proteins (five of which are reproductive tract-specific) have abundantly expressed protein in the HET testis compared to the KO testis. (**b**) NEK3 is more abundantly expressed in KO testis compared to HET testis.

Multiple proteins from the proteomics profiling, in addition to PRSS37, were found to have higher abundance in the HET mice compared to the KO mice (Fig. 6a). Of these proteins, it is important to note that five of the ADAM proteins follow a similar pattern in the presence of PRSS37. ADAM3, ADAM4, ADAM5 and ADAM6(A&B) showed higher expression in the HET compared to the KO, which follows since ADAM4, ADAM5 and ADAM6 are known to form trimeric complexes with ADAM2-ADAM3^11^. These complexes are known to be important in sperm migration through the UTJ, and since ADAM3 is depleted in PRSS37 KO mice, it also confirms our findings that other proteins associated with the trimeric complexes are also depleted since PRSS37 has been suggested as a protein chaperone^6–9,11–14^. Additionally, other proteins found to be more abundantly expressed in HETs than in KOs, such as SLXL1^42^, SPATA20^43,44^, and TRIM36^45^, have all been shown to be essential for male fertility.

One interesting piece of data that came from our proteomics research was an upregulation of NEK3 in the KO mice compared to the HET mice (Fig. 6b). NEK3 is important in mitotically active tissue, such as testicular germ cells^46^; and the increased abundance of NEK3 in KO mice is surprising since sperm morphologically are normal and only have an issue traversing the UTJ.

We examined this parameter in the TGCs and in the corpus epididymis since that tissue had the most differentially expressed proteins. We found 5 proteins that were exclusively expressed in male reproductive tissue and protein abundance was differentially expressed between the PRSS37 HET and KO in the corpus epididymis (Fig. 5a). AKAP4, is first present during spermatogenesis and then is later cleaved on spermatozoa to help with sperm motility^47^. PGK2 is also important in the function of the flagella to help with motility since it is essential for providing glycolytic ATP production^48^. LDHC is another enzyme that is essential for the glycolysis pathway; sperm that lack LDHC are unable to achieve hyperactivation or bind to the ZP^48^. A disruption in GK2 will result in a malformation of mitochondria in the sperm cell and as a result sperm are unable to pass through the UTJ^49^. One possible explanation for these observed decrease in protein expression could be a direct or indirect result of loss of PRSS37. Our research has demonstrated that multiple proteins are disrupted in the absence of PRSS37, whether that be from lack of the protein itself, how it regulates other cascades (including protein processing), and/or through a stabilizing role of PRSS37 in a multi-protein complex on the sperm surface. As well might be the case for TMPRSS12, since we know it functions in conjunction with ADAM3 and also has regulatory effects over sperm motility and migration through the UTJ^40^. There are other proteins whose abundance matches a pattern of expression similar to PRSS37 but are not on their own essential for male fertility such as DNAJB8^50^, which may indicate the need for a larger cascade or dual loss for sufficient fertility suppression. Further examination of these proteins is required to determine if they would be suitable candidates for non-hormonal male contraception.

We acknowledge that several limitations exist in this research. We used HET mice instead of WT mice as our control since the HETs reproducibility was similar to the WT mice. While we used a mouse model to complete these experiments, further research needs to be completed on additional human tissues in an in vitro model. Additionally, while we used a transgenic animal that had a humanized protein, there could be potential disruptions or differences in downstream protein cascades that could explain why we only observed a partial rescue in our transgenic mice.

Our data is the first to show that the infertile phenotype presented by* Prss37* KO mice can be rescued using a humanized transgenic* Prss37* rescue mouse. It also shows that other serine protease production may also be impacted by deletion of PRSS37. Our research confirms some of the findings from others^6,7^ and also provides new insights and targets for further exploration as viable compounds for contraceptive development. Further research needs to be done to prepare a drug compound to suppress PRSS37 function and can then be moved into clinical trials for contraception development.

In conclusion, our studies using *Prss37* KO mice and proteomics confirm that PRSS37 is essential for male fertility and demonstrate that fertility can be rescued using a humanized PRSS37 protein in a transgenic mouse model. These findings further validate the pursuit of PRSS37 as a target for non-hormonal male contraception.

### Supplementary Information

Supplementary Information 1.

Supplementary Information 2.

## Data Availability

The data that support the findings of this study are openly available at Computer Science and Engineering University of California, San Diego Center for Computational Mass Spectrometry (MassIVE: https://massive.ucsd.edu/ProteoSAFe/static/massive.jsp) with the following accession number: MSV000091662.
