# Supplementary material for: Molecular dissection and testing of PRSS37 function through LC–MS/MS and the generation of a PRSS37 humanized mouse model
Source: Sci Rep. 2023 Jul 14;13:11374. doi: 10.1038/s41598-023-37700-1 (PMC10349139; doi:10.1038/s41598-023-37700-1)
Supplement: Supplementary file 1 — Supplementary Information 1. [file 41598_2023_37700_MOESM1_ESM.pdf]

Supplementary Figures

Molecular dissection and testing of PRSS37 function through LC-MS/MS and the generation of a PRSS37 humanized mouse model

Fig S1. Creating PRSS37 mouse lines

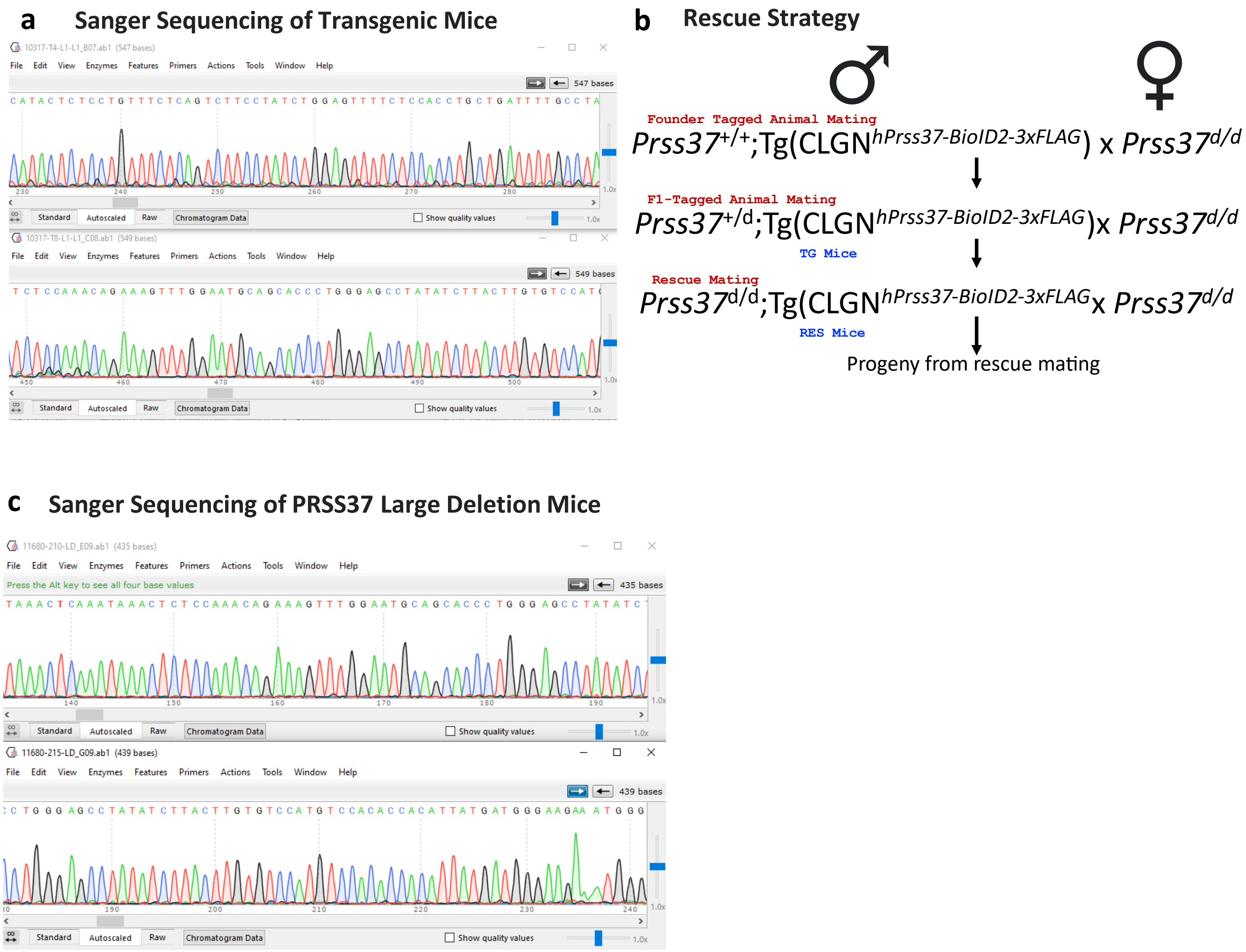

Figure S1. Creating PRSS37 mouse lines

a. Sanger sequencing for *Prss37* TG mice

b. Rescue strategy for developing RES mice from the transgenic mice produced at Baylor College of Medicine.

c. Sanger sequencing for *Prss37* KO large deletion mice.

Fig S2. CASA results from *Prss37* HET, KO, TG and RES mice

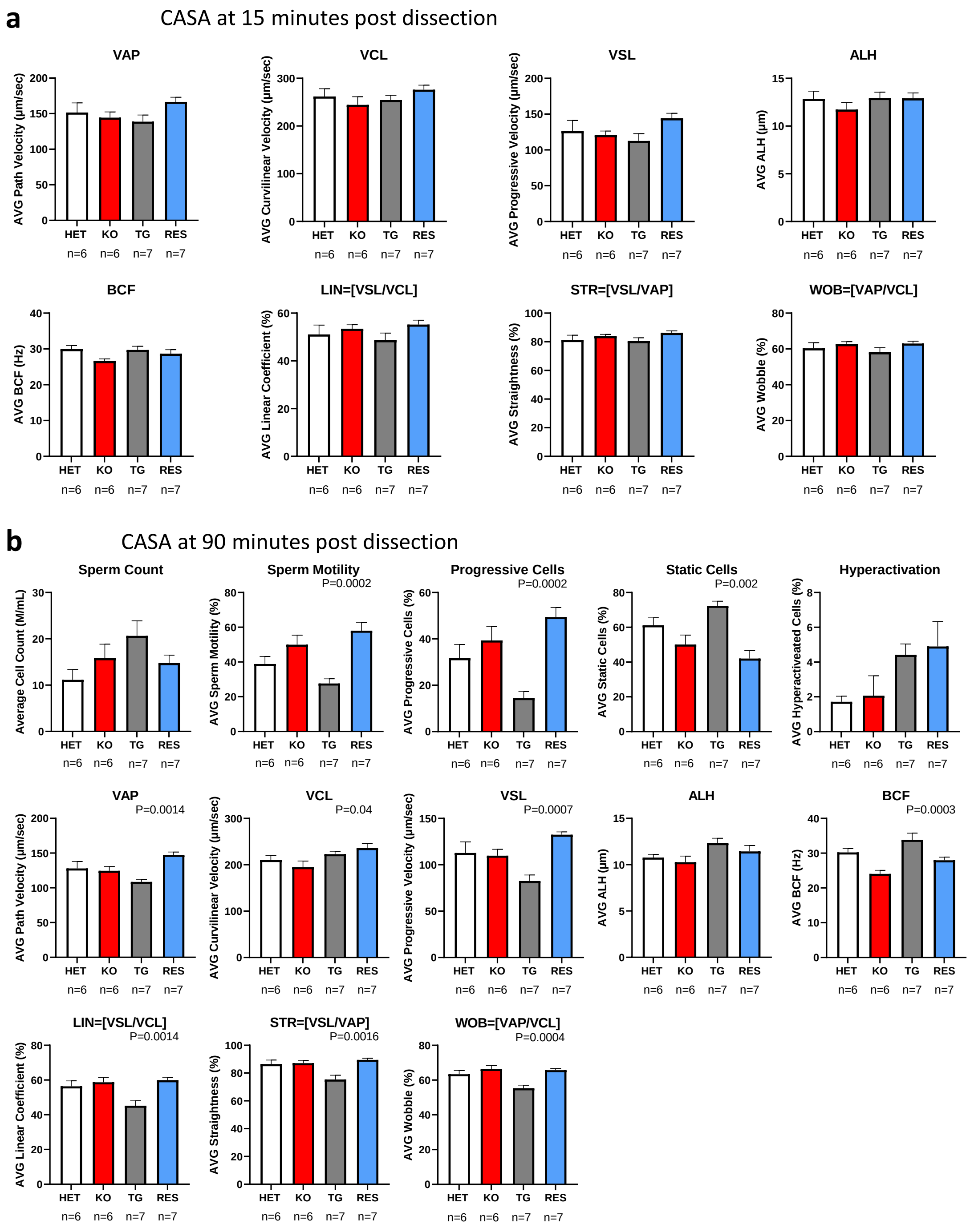

Figure S2. CASA results from *Prss37* HET, KO, TG and RES mice

a. CASA parameters between PRSS37 HET, KO, TG, and RES mice at 15 minutes post dissection.

b. CASA parameters between PRSS37 HET, KO, TG, and RES mice at 90 minutes post dissection.

Fig S3. Expression of proteins of interest by tissue type

a. Testicular Germ Cells

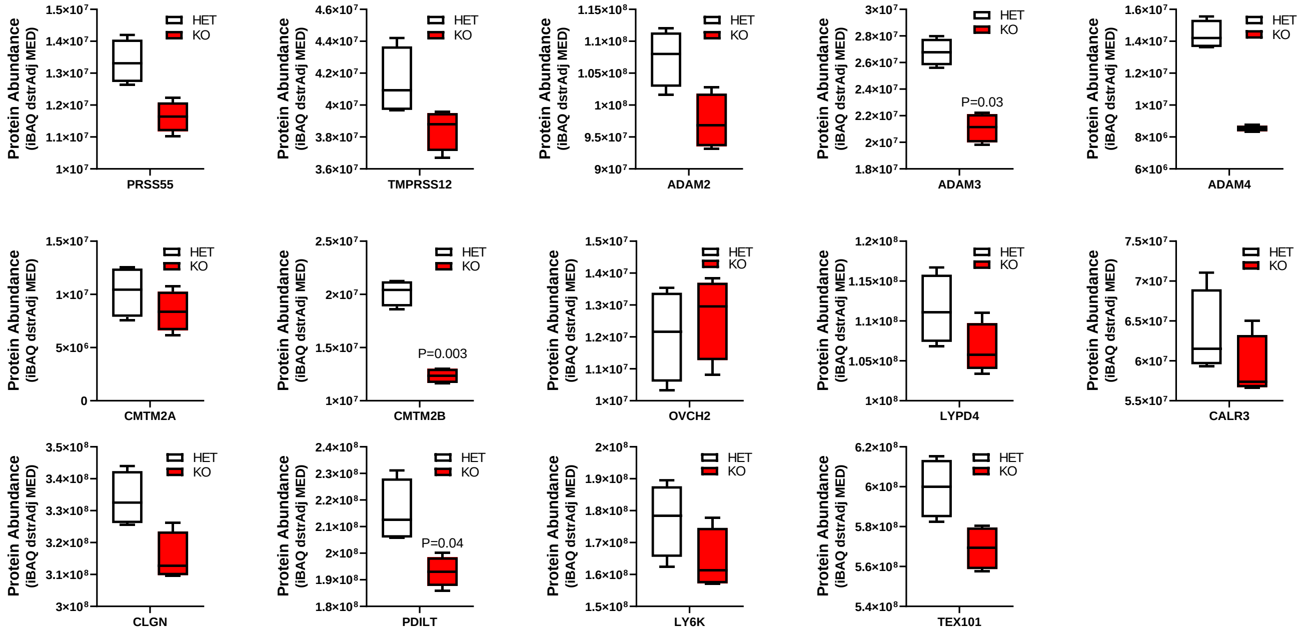

b. Caput Epididymis

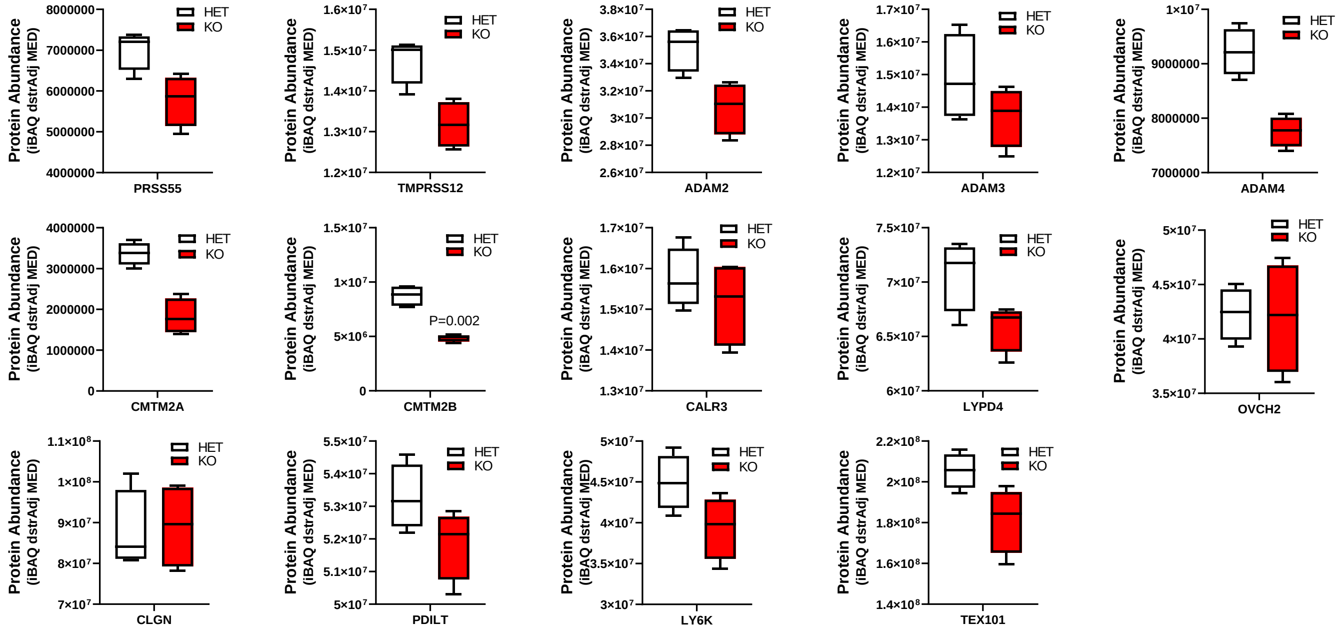

Figure S3. Expression of proteins of interest by tissue type

a. Protein of interest abundance in testicular germ cells.

b. Protein of interest abundance in the caput epididymis.

c. Protein of interest abundance in the corpus epididymis.

d. Protein of interest abundance in the cauda epididymis.

Fig S3(cont). Expression of proteins of interest by tissue type

c. Corpus Epididymis

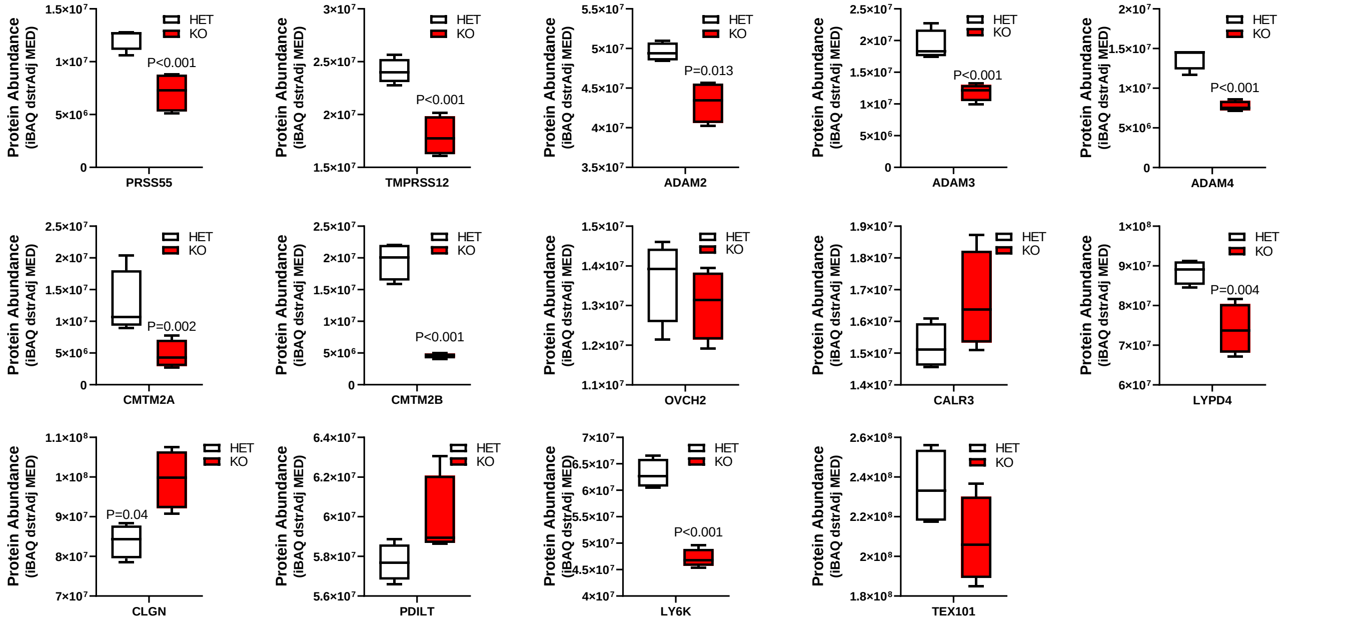

d. Cauda Epididymis

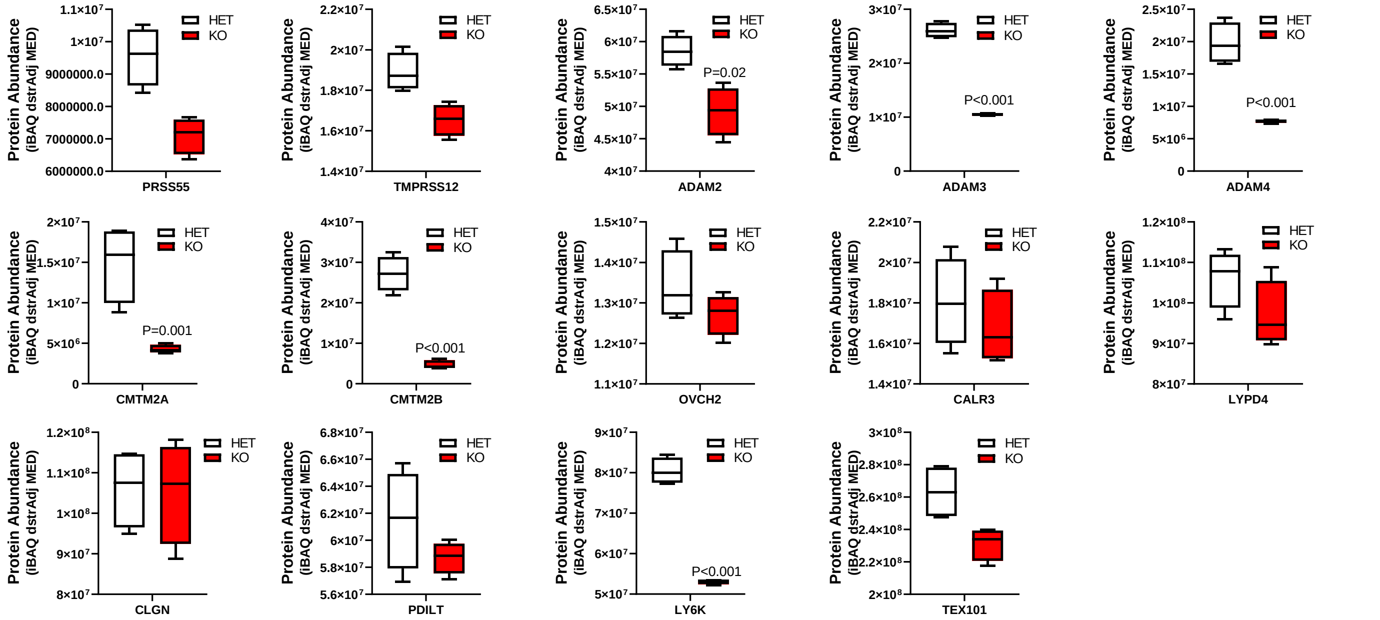

Figure S3. Expression of proteins of interest by tissue type

a. Protein of interest abundance in testicular germ cells.

b. Protein of interest abundance in the caput epididymis.

c. Protein of interest abundance in the corpus epididymis.

d. Protein of interest abundance in the cauda epididymis.

Fig S4. Uncut/cropped gels and blots

a.

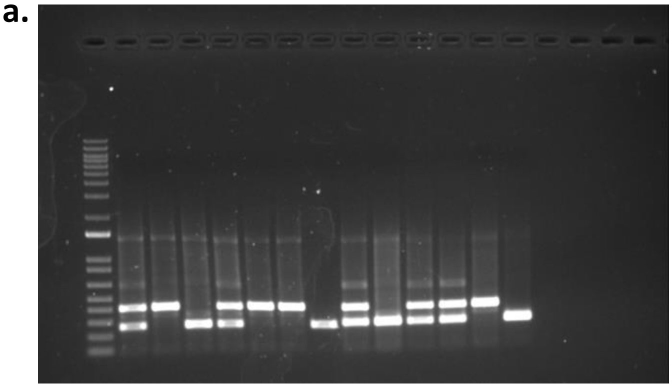

b.

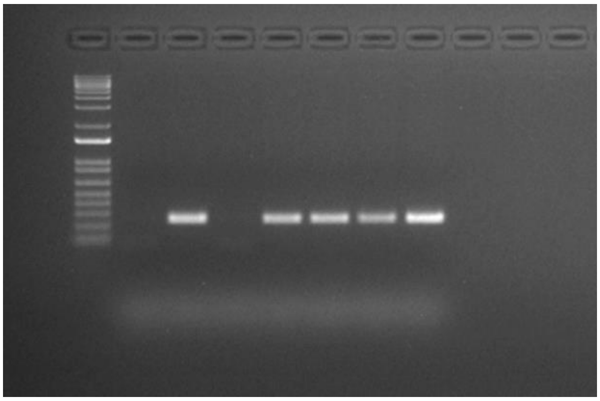

c.

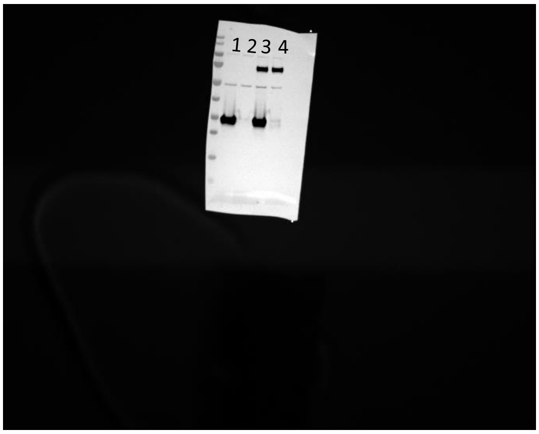

d.

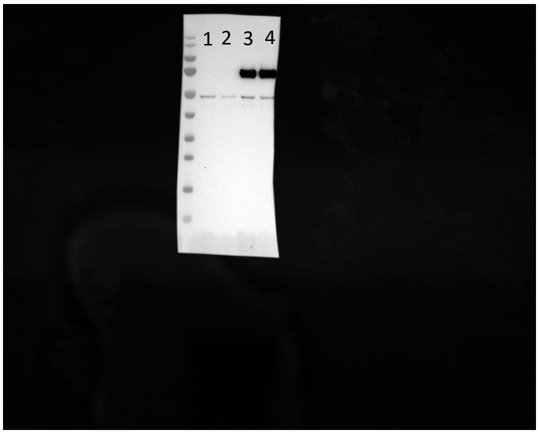

e.

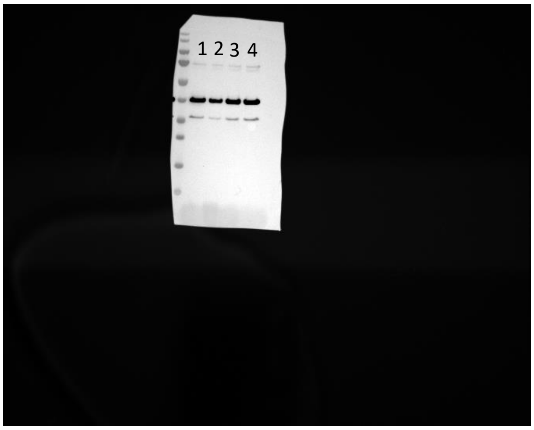

**1. HET**	*Prss37* $^{+/d}$
**2. KO**	*Prss37* $^{d/d}$
**3. TG**	*Prss37*$^{+/d}$;Tg(CLGN$^{hPrss37\text{-}BioID2\text{-}3xFLAG}$)
**4. RES**	*Prss37*$^{d/d}$;Tg(CLGN$^{hPrss37\text{-}BioID2\text{-}3xFLAG}$)

Figure S4. Uncut/cropped gels and blots

a. Gel of *Prss37* genotyping; HET, WT, KO control are in the last 3 wells, respectively.

b. Gel for *hPrss37* transgenic mice genotype; positive control is the last well.

c. Blot of Anti-PRSS37 HET, KO, TG, and RES in lanes 1-4, respectively.

d. Blot of Anti-FLAG HET, KO, TG, and RES in lanes 1-4, respectively.

e. Blot of Anti-GAPDH HET, KO, TG, and RES in lanes 1-4, respectively.

Fig S5. Blots for ADAM3 that were different between HET and KO mice

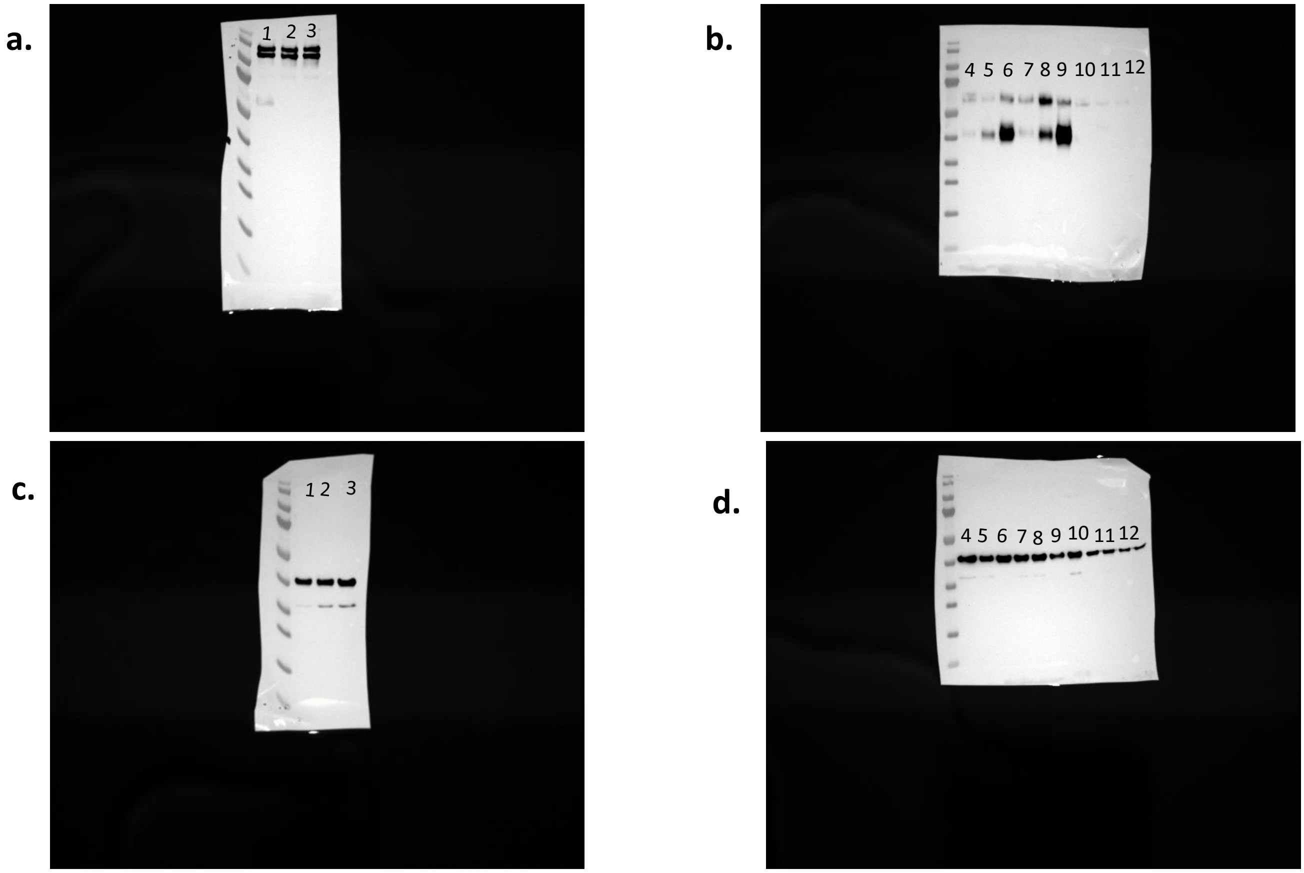

KEY FOR BLOTS

1. WT Testis
2. HET Testis
3. KO Testis
4. WT Caput
5. WT Corpus
6. WT Cauda
7. HET Caput
8. HET Corpus
9. HET Cauda
10. KO Caput
11. KO Corpus
12. KO Cauda

Figure S5. Blots and gene expression for genes that were different between HET and KO mice

a. Blot of Anti-ADAM3 in the testis tissue

b. Blot of Anti-ADAM3 in the epididymis tissue

c. Blot of Anti-GAPDH in the testis tissue

d. Blot of Anti-GAPDH in the epididymis tissue*

*There are dual lanes for 11 and 12 that have the same sample run side by side.

Fig S6. Gene expression for genes that were different in the proteomics data between HET and KO mice

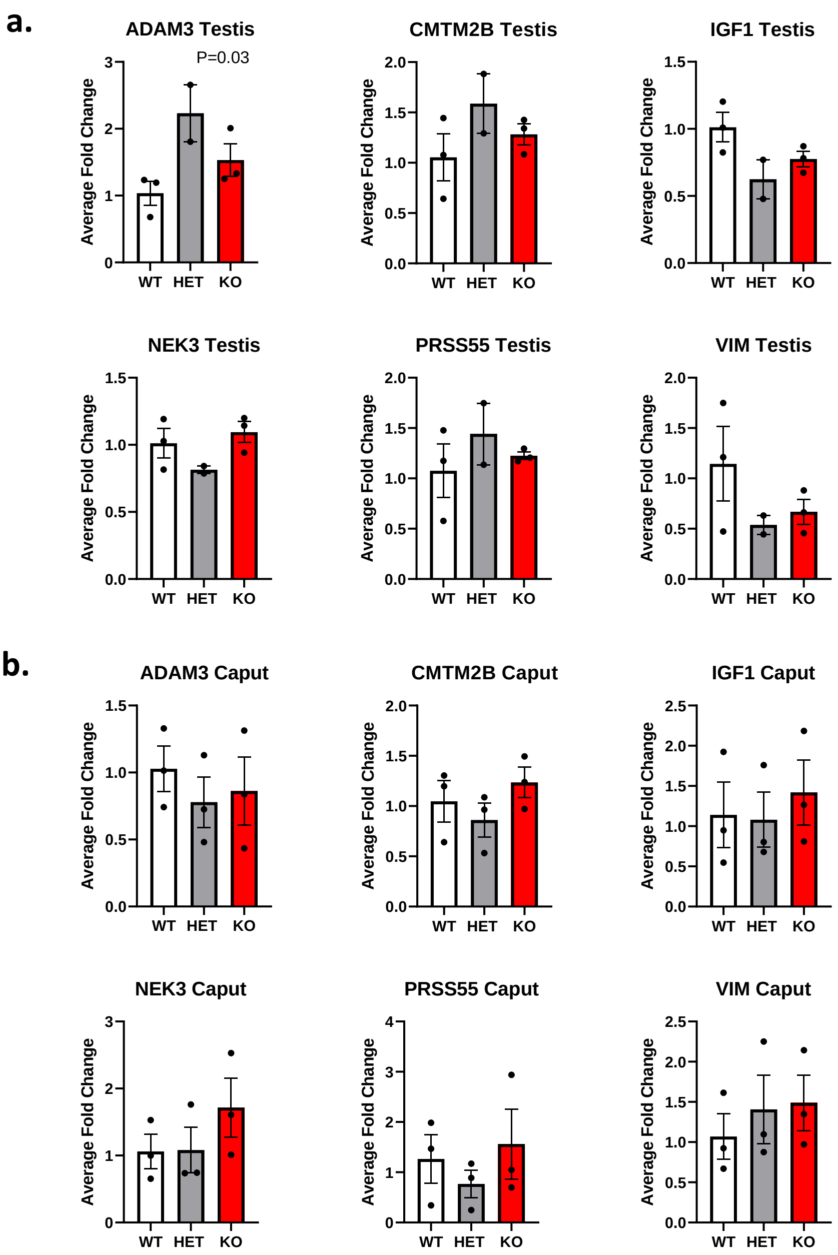

Figure S6. Gene expression for genes that were different in the proteomics data between HET and KO mice

a. Average fold change of six genes in the testis tissue. Using TaqMan Gene Expression Assay Mm00442269_m1, Mm01250968_m1, Mm00456455_m1, Mm01313036_m1, Mm01333430_m1, and Mm00439560_m1.

b. Average fold change of six genes in the caput tissue Using TaqMan Gene Expression Assay Mm00442269_m1, Mm01250968_m1, Mm00456455_m1, Mm01313036_m1, Mm01333430_m1, and Mm00439560_m1.

Fig S7. Protein down/up regulation in *Prss37* KO mice

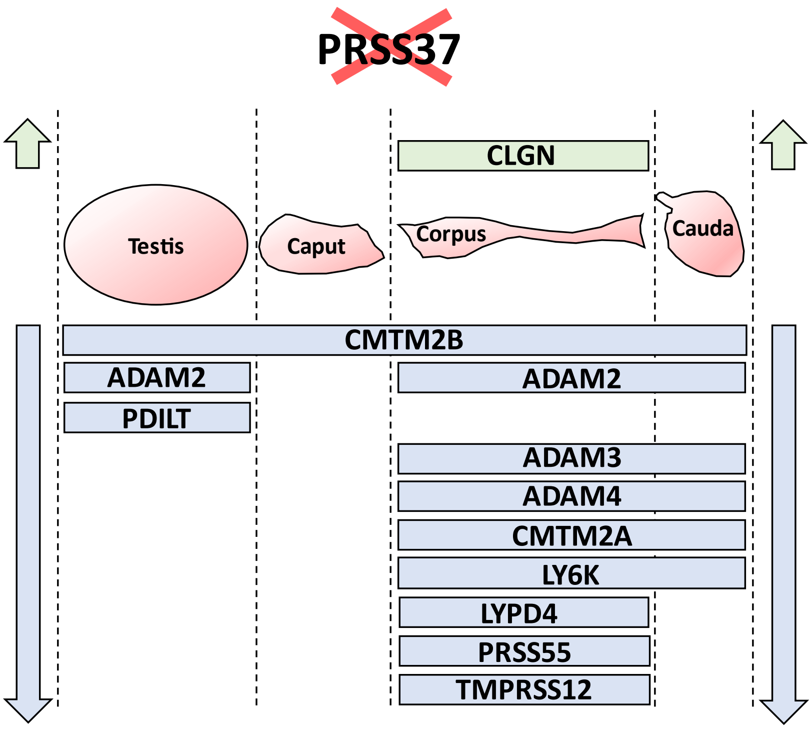

Figure S7. Protein down/up regulation in Prss37 KO mice

Figure showing several proteins of interest that are down regulated or up regulated in the KO mouse based on our proteomic data.

Supplementary Table Legend

Table S1. Proteomics Profiling Results

Each bulleted letter below indicates a separate sheet.

a. Concatenated Final Output
    a. This table includes all analyzed data in Gene ID order with gene symbol and brief description included. Data are sorted between HET vs KO by tissue as well as Tissue vs Tissue. Peptide counts and Original iBAQ dstrAdj_MED values are included as well.

b. All of the following sheets contain this information: This table has Gene ID in the first column followed by the log2 fold change with right and left confidence intervals, then the p-value and adjusted p-value are given. All of these data are generated from columns J-AO which correspond to selected tissue/treatment proteomics values that have been normalized by using a log10 transformation. The gene symbols and description follow those values in columns AP-AS.
    a. The list of sheets include:
        a. HET vs KO Testis
        b. HET vs KO Caput
        c. HET vs KO Corpus
        d. HET vs KO Cauda
        e. Caput vs Testis
        f. Cauda vs Caput
        g. Cauda vs Corpus
        h. Cauda vs Testis
        i. Corpus vs Caput
        j. Corpus vs Testis
